# Neuroprotective effects of an engineered *Escherichia coli* Nissle 1917 on Parkinson's disease in mice by delivering GLP‐1 and modulating gut microbiota

**DOI:** 10.1002/btm2.10351

**Published:** 2022-06-18

**Authors:** Heng Wu, Jing Wei, Xiumiao Zhao, Ying Liu, Zhihang Chen, Kehong Wei, Jiachen Lu, Wenjie Chen, Meixiu Jiang, Shengjie Li, Tingtao Chen

**Affiliations:** ^1^ National Engineering Research Center for Bioengineering Drugs and the Technologies, Institute of Translational Medicine Nanchang University Nanchang Jiangxi China; ^2^ Queen Mary School Nanchang University Nanchang Jiangxi China; ^3^ Institute of Life Science Nanchang University Nanchang Jiangxi China

**Keywords:** *E. coli* Nissle 1917, genetically engineered strain, GLP‐1, gut microbiota, Parkinson's disease

## Abstract

Considerable evidence suggests that insulin resistance is closely linked to Parkinson's disease (PD), leading to agents aiming at treating diabetes can be regarded as new neuroprotective strategies in PD, notably glucagon‐like peptide‐1 (GLP‐1). However, the extremely short half‐life of GLP‐1 due to degradation by the ubiquitous proteolytic enzyme limits its clinical application. In this study, we engineered the recombinant integrant probiotic strain *Escherichia coli* Nissle 1917 (EcN) to create a strain EcN‐GLP‐1 that effectively delivers the heterologous GLP‐1 molecule. Subsequently, we assessed its neuroprotective effects on 1‐methyl‐4‐phenyl‐1, 2, 3, 6‐tetrahydropyridine (MPTP)‐induced PD mice. We demonstrated that EcN‐GLP‐1 treatment could improve motor deficits, increase tyrosine hydroxylase‐positive neurons, suppress microglia and astrocyte activation, reduce brain and colon inflammation, and ameliorate colonic barrier function damaged by MPTP induction. Meanwhile, we confirmed that the oral administration of EcN‐GLP‐1 could restore the disturbance of gut microbiota in the MPTP‐induced PD mice, by reducing the relative abundances of *Akkermansia* and *Oscillospira*, and increasing the level of *Prevotella* in the gut. These results support further development of an engineered probiotic platform in which production of GLP‐1 for gut‐brain disorders, such as PD.

## 1 INTRODUCTION

Parkinson's disease (PD) is a common progressive neurodegenerative disease exhibiting several primary motor symptoms, including tremor, rigidity, gait instability, bradykinesia, and impairment of postural reflex, as well as a series of nonmotor symptoms, such as sleep deficits, cognitive impairment, depression, and even intestinal dysfunction.^1^, ^2^, ^3^ As a multifactorial disease, PD is typically featured by the progressive loss of dopaminergic neurons in substantia nigra pars compacta (SN), and the abnormal accumulation of α‐synuclein (α‐Syn) that forms Lewy bodies and Lewy neuritis.^4^, ^5^ Several studies have indicated that the onset and progression of PD are caused by the dysfunction of multiple cellular pathways, including mitochondrial functional impairment, dysregulation of calcium homeostasis, excitotoxicity, endoplasmic reticulum stress, and oxidative stress, resulting from the aggregation of various pathological proteins.^6^, ^7^ Meanwhile, neuroinflammation accompanied by overexpression of multiple pro‐inflammatory cytokines, for example, interleukin‐1β (IL‐1β), IL‐6, and tumor necrosis factor‐α (TNF‐α), is also critical in the pathogenesis of PD.^8^, ^9^

In our previous work, we emphasized that the gut microbiota and its metabolic profiles are involved in the occurrence, development, and treatment of neurodegeneration disorders via the microbiota‐gut‐brain axis, indicating that intestinal dysfunctions may contribute to the pathogenesis of PD.^10^ Several studies also demonstrated that the gut microbiome, including its composition and structure, and microbial metabolites, are significantly altered in PD patients and animal models.^7^, ^11^ As we mentioned, PD is caused by the abnormal formation of α‐Syn, which had been observed to ascend from the intestine to the brain, supporting the intestinal origin of PD.^12^ Recent studies have suggested that α‐Syn can be expressed by several enteroendocrine cell subtypes and transmitted to connected enteric nerves, leading to a reasonable hypothesis that intestinal inflammation facilitates the pathological aggregation of α‐Syn in the brain, which then contributes to the pathogenesis of PD.^13^, ^14^ Meanwhile, increased intestinal permeability in PD is accompanied by reduced expression of crucial tight junction proteins (e.g., ocludin), which can disrupt the intestinal barrier integrity and lead to the accumulation of α‐Syn in the gut and brain.^15^ Therefore, new insights into gut and gut microbiota‐targeted strategies will provide novel ways to treat PD.

At present, several compounds and strategies have been used as the first‐line treatments to prevent PD or halt its progression, including dopamine precursor levodopa, dopamine agonists, anticholinergics, and other inhibitors (e.g., catechol‐o‐methyl transferase inhibitors and monoamine oxidase B inhibitors). However, they clinically failed to slow the neurodegenerative process of PD and even exert serious side effects.^7^ Moreover, probiotics/prebiotics (so‐called psychobiotics) and fecal microbiota transplantation have been considered the most promising therapeutic interventions in PD nowadays.^16^, ^17^ Nevertheless, further understanding of the roles and mechanisms of those interventions in treating PD is still needed, since the fundamental research on which enteric bacteria communicate with the brain and how the gut microbiota causally influences the brain is inadequate.^10^ Therefore, exploring therapeutic interventions in halting PD is still in demand.

Clinical and epidemiological studies have demonstrated the links between diabetes and the pathogenesis of PD, particularly reminding us that various agents in treating diabetes might have unexpected neuroprotective actions in PD.^6^ Glucagon‐like peptide‐1 (GLP‐1), a prototype of enteroglycemic physiologically released by enteroendocrine L‐cells, has been indicated to be essential for glucose homeostasis and is used to treat diabetes in response to food intake and metabolism modulation.^18^, ^19^ GLP‐1 reduces postprandial hyperglycemia with an extremely short half‐life due to degradation by the ubiquitous proteolytic enzyme (mainly dipeptidyl peptidase‐4 [DPP‐4] in the plasma).^20^ Therefore, replenishing persistently exogenous GLP‐1 to avoid the proteolysis of DPP‐4 or developing its analogs are better strategies for prolonging the biological efficacy of GLP‐1. Our previous studies have suggested that the engineered probiotic strains with plasmids encoding GLP‐1 have positive effects on diabetes and obesity.^21^, ^22^ Specifically, our previous studies also demonstrated that the GLP‐1‐delivering bacteria have an efficacious therapeutic effect on neuropsychiatric diseases, including PD in animal models.^23^, ^24^, ^25^ However, as plasmids may be lost in the absence of selection pressure or be unequally segregated during cell division, plasmid devices face genetic instability in practical situations.^26^

*Escherichia coli* Nissle 1917 (EcN) is commonly used as a treatment for human diseases due to its probiotic characteristics and robustness in the gastrointestinal tract. Sufficient genetic manipulation tools are suitable for EcN engineering, making it be the most widely used chassis for biomedical applications. Therefore, the administration of GLP‐1 combined with EcN may exhibit synergy for enhanced therapeutic efficacy in PD. In this study, we employed the CRISPR/Cas9 two‐plasmid system to integrate a DNA fragment containing GLP‐1 cluster genes with *hce* promotor and *pelB* signal peptide sequence into the *attB* loci in the chromosome of EcN,^27^ and acquired a resistance marker‐free genetically engineered strain EcN‐GLP‐1. We confirmed that EcN‐GLP‐1 efficiently produced GLP‐1 without affecting its probiotic properties, including growth ability, acid and bile salt resistance, and adherence capacity. Then, we evaluated the neuroprotective effects of EcN‐GLP‐1 treatment on the 1‐methyl‐4‐phenyl‐1, 2, 3, 6‐tetrahydropyridine (MPTP)‐induced PD mice. The motor functions were assessed by the pole test and open field test. Meanwhile, the correlated protein/cytokines levels involved in the brain and colon inflammation, microglia and astrocyte activation, tyrosine hydroxylase‐positive neurons, as well as the colonic barrier function were assessed by Western‐blot, RT‐qPCR, immunohistochemistry (IHC), and immunofluorescence (IF). Gut microbial compositions were performed by 16S rRNA sequencing combined with bioinformatic analysis. These data support effective treatments based on GLP‐1 engineered probiotic strains targeting the intestinal tract to modulate PD.

## 2 MATERIALS AND METHODS

### 2.1 Plasmids, bacterial strains, and cell culture conditions

The bacterial strains and plasmids used in the present study are given in Table S1. EcN and its genetic engineering strain were grown in Luria–Bertani (LB) broth or on LB agar plates supplemented with appropriate antibiotics and cultured at 37°C or 30°C or 42°C with aeration as requested. *Staphylococcus aureus*, *Salmonella typhimurium*, and *Salmonella enteritidis* were grown in LB medium or on LB agar plates at 37°C with aeration. The mouse colon cancer cell line CT26 was cultivated in RPMI 1640 supplemented with 10% fetal bovine serum (FBS) and maintained in an atmosphere of 5% CO_2_ at 37°C.

### 2.2 Genetic engineering strain construction

All primers and the N20 sequence followed by the PAM in the target loci of *attB* ^28^ used in this study are listed in Table S2. *TransStart*® *FastPfu* DNA Polymerases (Transgene, AP211, Beijing, China) were employed in the PCR reaction. The two‐plasmid‐mediated CRISPR‐Cas9 system was selected to integrate the cluster genes into the genome of EcN as previously described.^27^

The pTargetT‐*attB*::P_hce_‐pelB‐glp1 recombinant plasmid was constructed as follows. First, the primers P1/P2 were used to linearize the pTargetF plasmid (Addgene, 62226) to form the pTargetF‐*attB* fragment containing the N20 sequence, and then the linearized amplicon was cyclized by using the ClonExpress® II One Step Cloning kit (Vazyme, C112, Nanjing, China), and the cyclized product was subsequently transformed into *Trans*5α competent cells to obtain a new recombinant plasmid pTargetF‐*attB*. Second, the upstream and downstream homologous arms of *attB* gene were PCR‐amplified by using the primers P3/P4 and P7/P8. The codon‐optimized *GLP‐1* gene clustered with HCE promotor^29^ and pelB signal peptide sequences^30^ was PCR‐amplified from the pBSC‐pelb‐hglp plasmid by using P5/P6 primers. The three amplicons above were purified and fused by overlapped PCR using P3/P8 primers, and then confirmed by 1% agarose gel electrophoresis and DNA sequencing. Third, using the above Cloning kit to fuse the tripartite fragment into the pTargetF‐*attB* linearized by P9/P10 primers in advance, and then transformed into the *Trans*5α competent cells to construct the recombinant plasmid pTargetT‐*attB*::P_hce_‐pelB‐glp1.

The recombinant integration strain was constructed as previously described.^31^ In brief, the pCas plasmid (Addgene, 62225) was firstly transformed into EcN, and then cultured with 10 mM arabinose to prepare competent cells. The pTargetT‐*attB*::P_hce_‐pelB‐glp1 plasmid was transformed into the above prepared EcN competent cells. After recovery at 30°C for 60 min, the cultures were spread onto the LB agar plates containing 50 μg/ml kanamycin and 50 μg/ml spectinomycin, and then grown at 30°C for overnight. The transformant clones were identified by colony PCR with primers P11/P12, and then the correct integrant strains were further confirmed by DNA sequencing. After obtaining the integrant strain, the pTargetT‐*attB*::P_hce_‐pelB‐glp1 was eliminated by culturing strains with 0.5 mM IPTG and 50 μg/ml kanamycin alone at 30°C for over 14 h, and then the pCas plasmid was finally removed by culturing the strains at 42°C overnight in LB medium. The recombinant integration strain was named EcN‐GLP‐1.

### 2.3 GLP‐1 protein expression, growth curve test, and probiotic properties assessment

The expression of GLP‐1 in the EcN‐GLP‐1 strain was verified by Human GLP‐1 ELISA kit (Invitrogen, EH221RB). EcN and EcN‐GLP‐1 strains were cultured in 200‐ or 300 ml LB medium with shaking (180 rpm) at 37°C for 24 h; 1 ml cultures were harvested by centrifuging (10,000 g, 5 min, 4°C) and the cell‐free supernatant was collected for ELISA assay according to the kit's protocol. Growth curve was performed as follows. Briefly, EcN and EcN‐GLP‐1 colonies were grown in 5 ml LB medium to OD_600_ ≈ 2.0 and chilled on ice. To map the growth curve, 2.0 ml aliquots were resuspended in 200 ml fresh LB medium and incubated at 37°C with shaking at 180 rpm. The OD_600_ was monitored every 2 h for a total incubation period of 24 h. The growth curves were created from at least three independent measurements using GraphPad Prism.

Probiotic properties, including acid resistance, bile salt resistance, adherence capacity, antioxidant, and antibacterial capacity, were assessed as previously described with slight modifications.^32^ For acid resistance test, the bacterial sediment was collected by centrifugation, resuspended in sterile PBS, and then diluted in PBS with various pH values, including 2, 3, 4, 5, 7. The cell suspension was incubated at 37°C for 4 h, and the live bacterial number was calculated by the viable counting method. For bile salt tolerance, equal amounts of bacteria were added into the LB medium containing varying concentrations of bile salt (0.0%–0.5%), and then incubated at 37°C for 4 h. The viable count method was performed to detect the viable bacteria.

The cell‐free supernatant collected above was used to assess the antioxidant and antibacterial activities. The clearance of DPPH, −OH, O_2_^−^, and Fe^2+^ chelation was selected to evaluate the antioxidant abilities of the two strains. The oxford cup method was carried out to detect the antibacterial activities, and the inhibition zones were measured after 4 h. For adherence capacity test, 10^8^ CFU bacteria were cocultured with CT26 cells for 1.5 h in six‐well cell culture plates that were pre‐placed with cover glasses. Then, the un‐adhered bacteria were removed by PBS washing, and the cover glasses were taken out and immobilized with methanol for 0.5 h. The adhered bacteria were stained by Gram staining, and the total amount of adhering bacteria was measured in 100 random CT26 cells. Each experiment was repeated three times.

### 2.4 Animals and experimental design

This study and the animal experiment protocol were viewed and approved by the Laboratory Animal Ethics Committee of Nanchang Royo Biotechnology Co., Ltd, Nanchang, China (Approval Number: RYE2020052001).

Sixty male C57BL/6 mice (28–32 g) were purchased from Hunan SJA Laboratory Animal Co., Ltd. (Changsha, Hunan, China), and housed in a standard animal room (temperature 24 ± 2°C, humidity 49 ± 11%, 12/12 dark–light cycle). Mice were allowed free access to water and a standard diet, and adapted to the environment for 1week. To reduce errors associated with the time of testing, all animal experiments were conducted between 8:30 and 11:30 am. Twelve mice were randomly selected and assigned to the normal control group (C group) injected intraperitoneally with saline. The remaining 48 mice were used to establish the PD animal model via injection intraperitoneally with 20 mg/kg/d MPTP (Sigma‐Aldrich, M0896) for 7 consecutive days. Unfortunately, nine mice died during the modeling process due to MPTP injection.

The residual members of the MPTP‐induced PD mice were randomly split into four groups: M group, *n* = 10; MN group, *n* = 9; ME group, *n* = 10; MG group, *n* = 10. Mice in the C and M groups were given 0.2 ml/day sterilized saline via gavage. Mice in the MN and ME groups were taken 0.2 ml/day saline containing 10^9^ CFU of EcN and EcN‐GLP‐1 via gavage, respectively. Mice in the MG group were intraperitoneally injected with 24 nM/kg/day Exenatide (an GLP‐1 receptor agonist indicated for revealing the effect and mechanism of GLP‐1 on PD, a positive control) that dissolved in saline. All the treatments were performed continuously for 7 days. The behavioral performance of each mouse was assessed by the pole test and open field test, and then sacrificed after intraperitoneal injection of 1% amobarbital sodium. Stool samples were harvested from the rectum and stored in 30% glycerol at −80°C for high‐throughput sequencing. Brain and colon tissues were collected after sacrifice and stored partly at −80°C and partly in 4% paraformaldehyde for subsequent experiments.

### 2.5 Pole test and open field test

The pole test and open field test were performed as we described previously.^23^, ^33^ Briefly, in the pole test, mice were placed facing upwards at the top of the rough wooden pole after training, and the time of each test trial was recorded. In the open field test, each mouse was individually placed in the field apparatus to adapt for 5 min, and then their behavioral routes were monitored for 5 min with the video tracking software. The equipment was thoroughly wiped with 75% alcohol to eliminate residual liquid and odor after each test.

### 2.6 Immunohistochemistry and immunofluorescence

IHC and IF experiments were performed following standard procedures. Brain tissue was immersed in 4% paraformaldehyde, cut into 5‐μm‐thick sections, de‐paraffinized in xylene, and rehydrated in a graded alcohol series. The sections were then incubated with 3% H_2_O_2_ for 5–10 min to block endogenous peroxidase activity and incubated with 5% goat serum for 1 h at room temperature. For IHC, sections were incubated with primary antibodies overnight at 4°C, including rabbit anti‐ionized calcium‐binding adaptor molecule‐1 (Iba1, CST, #17198), rabbit anti‐glial fibrillary acidic protein (GFAP, CST, #80788), rabbit anti‐α‐synuclein (α‐Syn, CST, #4179). For IF, rabbit anti‐tyrosine hydroxylase (TH, Proteintech, 25,859‐1‐AP) was used to the primary antibody. Cy3‐conjugated anti‐rabbit antibody (Servicebio, GB21303) was used as the secondary antibody.

### 2.7 Western‐blot analysis

Brain tissues and colon tissues were homogenized. Tissues were lysed by RIPA lysis solution containing protease inhibitors (Solarbio, China, R0010), and then proteins were obtained by centrifugation at 12,000 rpm for 30 min at 4°C. Protein concentrations were quantified by BCA kit (Thermo Fisher, A53226), then separated by SDS‐PAGE on 10%–12% gel and electro‐transferred to polyvinylidene difluoride membranes (Millipore, IPVH00010). After blocking with 5% skim milk at room temperature for 1 h, the membranes were then incubated with the specific primary antibodies, including rabbit anti‐GAPDH (CST, 174S), rabbit anti‐phosphorylated‐AKT (p‐AKT, CST, 5012S), rabbit anti‐AKT (CST, 4691S), rabbit anti‐phosphorylated‐IκB‐α (p‐IκB‐α, CST, 2859S); rabbit anti‐NF‐κB (CST, 8242S), rabbit anti‐phosphorylated‐NF‐κB (p‐NF‐κB, Abcam, ab86299), mouse anti‐toll‐like receptor 4 (TLR4, Santa Cruz Biotechnology, sc‐293072), rabbit anti‐zonula occludens‐1 (ZO‐1, Abcam, ab221547), and rabbit anti‐occludin (CST, 91131S). After washing more than three times, the membranes were incubated with goat anti‐rabbit antibody (Abcam, 205718) or goat anti‐mouse antibody (Abcam, 97265) for 2 h at room temperature. Finally, those specific proteins were detected by using a chemiluminescence solution (Thermo Fisher, 32209).

### 2.8 RNA preparation and RT‐qPCR

Total RNA was extracted from brain and colon tissues using TRIzol reagent according to the manufacturer's protocol (Invitrogen, 15596026). RNA degradation and contamination were assessed by 1% agarose gel. RNA concentrations were assayed by NanoDrop 2000 spectrophotometer (Thermo Fisher Scientific, Waltham, MA, USA). Genomic DNA was removed and then transcribed into cDNA using PrimeScript™ RT reagent Kit with gDNA Eraser (Takara, RR047A). The procedure of RT‐qPCR was carried out using TB Green Premix Ex Taq II kit (Takara, RR820A), and all reactions were performed using ABI 7500 Fast Real‐Time PCR System (ABI, Foster City, CA, USA). The program of RT‐qPCRs was 95°C for 30 s, followed by 40 cycles of 95°C for 5 s and then 60°C for 34 s. Primers for IL‐6, TNF‐α, IL‐1β, and GAPDH were listed in Table S2. The relative expression levels were measured using the 2^−ΔΔCt^ method.

### 2.9 High‐throughput sequencing analysis

The microbial genomic DNA was extracted according to the protocol of the DNA extraction kit (Tiangen, DP712). The quality and concentration of extracted genomic DNA were assessed by 1% agarose gel and NanoDrop 2000 spectrophotometer, respectively. Then, primers 515F/ 806R were used to amplify the V4 region of the 16S rDNA genes in each sample, and the products were sequenced with an Illumina NovaSeq6000 platform. Paired‐end reads from the original DNA fragments were processed using Cut adapt (version 1.9.1) and UCHIME Algorithm (http://www.drive5.com/usearch/manual/uchime_algo.html). The UPARSE package (Uparse v7.0.100, http://drive5.com/uparse) was then used for sequence analysis. Sequences with ≥97% similarity were assigned to the same operational classification unit (OTU). In‐house perl scripts were used to analyze β (between‐sample) diversity. Prior to the clustering analysis, a weighted UniFrac distance analysis was performed using the Quantitative Analysis of Microbial Ecology (QIIME) package and the metabolic capacity of the species found in different abundances was characterized by PICRUSt (version 1.0.0). The raw reads were deposited in the Sequence Read Archive (SRA) database of NCBI (PRJNA761145).

### 2.10 Data analysis

The Prism software version 7.0 (GraphPad Software, San Diego, CA, USA) was used for statistical analysis. Data are shown as mean ± SD. Analysis of statistical significance was performed using one‐way analysis of variance (ANOVA) followed by Tukey's multiple comparison test. *p* < 0.05 was regarded as statistically significant.

## 3 RESULTS

### 3.1 EcN‐GLP‐1strain was constructed and its probiotic properties were verified

**FIGURE 1:**
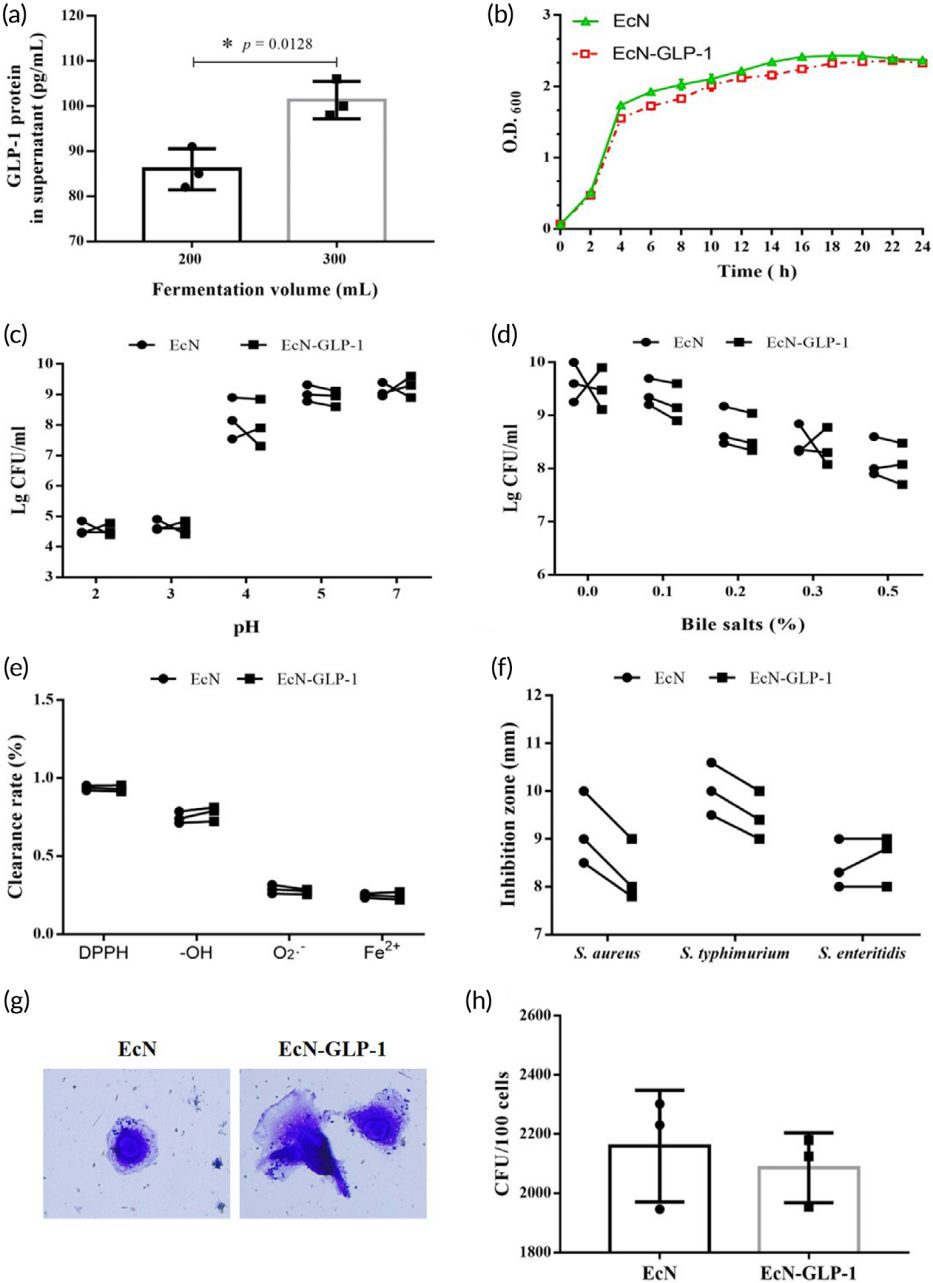
Expression of GLP‐1 in the engineered probiotic strain and evaluation its probiotic characteristics. (a) ELISA analysis of EcN‐GLP‐1 expressing and secreting GLP‐1. (b) Growth curves of EcN‐GLP‐1 and EcN. (c) Acid tolerance capacity of EcN‐GLP‐1 and EcN. (d) Bile salt resistance of EcN‐GLP‐1 and EcN. (e) Antioxidant property of EcN‐GLP‐1 and EcN. (f) Antibacterial activity of EcN‐GLP‐1 and EcN. (g) Representative diagram of EcN‐GLP‐1 and EcN adhesion CT26 cells. (h) The CFU of adherent bacteria per 100 CT26 cells. Means ± SD was calculated from three independent experiments. Compared with EcN, *p < 0.05, no asterisk indicates no significant difference (*p* > 0.05)

The recombinant integrant probiotic strain of EcN‐GLP‐1 was successfully reconstructed by using the CRISPR‐Cas9 two‐plasmid system (see Figure S1), and the integration of GLP‐1 cluster genes containing P_hce_ promotor and pelB signal peptide into the locus of *attB* in the chromosome of the EcN strain was verified by PCR reaction and DNA sequencing (Figure S2). The expression of GLP‐1 gene on the chromosome of EcN‐GLP‐1 was confirmed by ELISA assay. As shown in Figure 1a, the content of GLP‐1 in the fermentation supernatant of EcN‐GLP‐1 was 86.00 ± 4.58 pg/ml (200 ml system) and 101.33 ± 4.16 pg/ml (300 ml system) after cultured 24 h in identical laboratory conditions, respectively. Meanwhile, the integration of GLP‐1 gene cluster did not significantly affect cell growth under optimal conditions, although EcN‐GLP‐1 strain showed slightly lower growth ability between the late logarithmic period and stationary phase (Figure 1b). Through the probiotic properties assessments, EcN‐GLP‐1 strain could maintain its capacity of tolerance to acid and bile salt, antioxidant properties, antibacterial activity, and adherence ability to CT26 cells (Figure 1c–h). These results indicated that integrating GLP‐1 gene cluster into the genome of EcN strain could efficiently mediate the expression and secretion of GLP‐1 to the surrounding extracellular space without affecting its growth and probiotic properties.

### 3.2 EcN‐GLP‐1 improved motor coordination and reversed the pathological changes in MPTP‐induced PD mice

**FIGURE 2:**
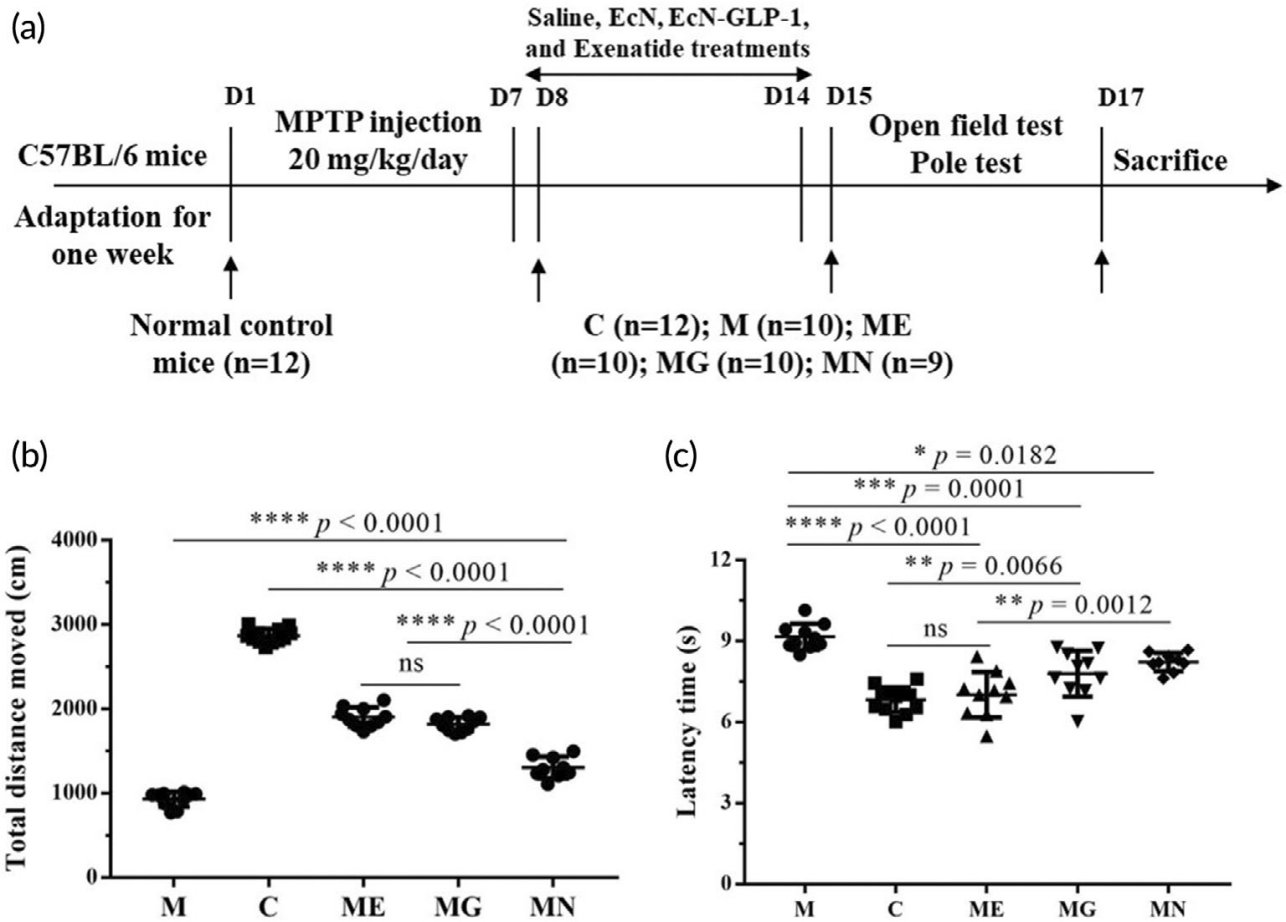
EcN‐GLP‐1 ameliorated the motor deficits in 1‐methyl‐4‐phenyl‐1, 2, 3, 6‐tetrahydropyridine (MPTP)‐induced Parkinson's disease (PD) mice. (a) The experimental scheme of this study. (b) EcN‐GLP‐1 increased the total moving distance of PD mice (open‐field test). (c) EcN‐GLP‐1 improved the bradykinesia of PD mice (pole test). C group, the normal control mice were treated with sterilized saline (*n* = 12); M group, the MPTP‐induced PD mice were treated with sterilized saline (*n* = 10); ME group, the MPTP‐induced PD mice that were orally taken EcN‐GLP‐1 (*n* = 10); MG group, the MPTP‐induced PD model mice were intraperitoneally injected with exenatide (*n* = 10); MN group, the MPTP‐induced PD mice were given EcN (*n* = 9). Data were presented as means ± SD. Tukey's multiple comparisons test, *p < 0.05, **p < 0.01, ***p < 0.001, ****p < 0.0001, ns indicates no significant difference (*p* > 0.05)

The animal experimental scheme is shown in Figure 2a. To observe the effects of EcN‐GLP‐1 on the locomotor coordination ability of PD mice, each mouse underwent both the open field and pole tests. In the open field experiments (Figure 2b), MPTP‐induced PD mice showed an obvious shorter total moving distance than that of normal control mice (930.40 ± 89.24 vs. 2867.33 ± 83.16 cm). However, this parameter was improved moderately by the treatment of EcN‐GLP‐1 (1940.90 ± 117.21 cm), EcN (1306.22 ± 127.99 cm), and exenatide (1820.3 ± 81.39 cm). Besides, EcN‐GLP‐1 showed a higher efficacy against MPTP‐induced PD than that of EcN strain, and a similar therapeutic effect with exenatide. The results of pole climbing test suggested that EcN‐GLP‐1 showed the same tendency as that of the open field test (Figure 2c). The induction of MPTP resulted in marked motor retardation in the PD model mice compared with C group (9.16 ± 0.48 s vs. 6.98 ± 0.48 s), which could be ameliorated significantly by treating with EcN‐GLP‐1 (7.13 ± 0.84 s). Collectively, these results suggested that the treatment of EcN‐GLP‐1 could restore the motion behavior parameters in MPTP‐induced PD mice.

**FIGURE 3:**
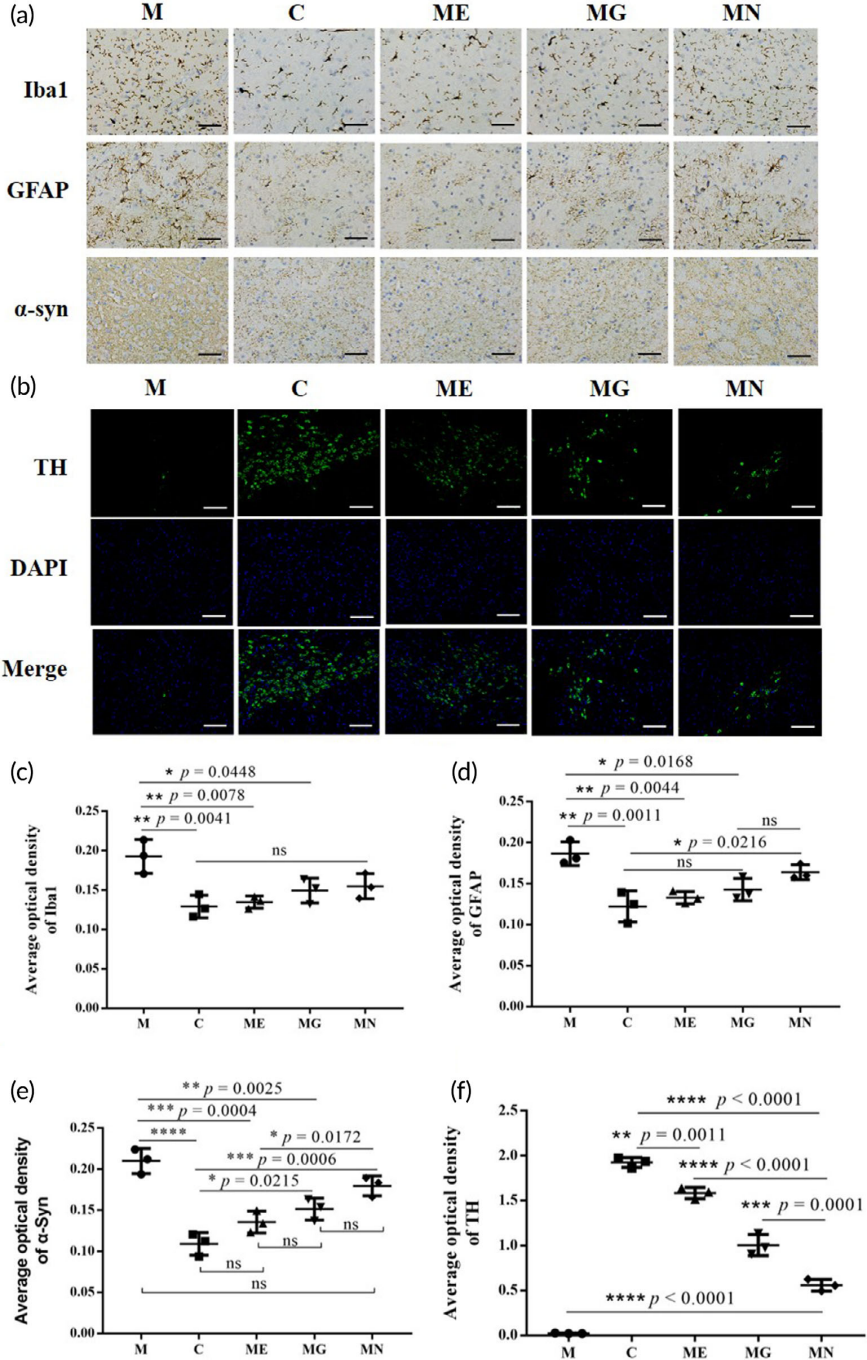
EcN‐GLP‐1 improved the neuropathologic damages in 1‐methyl‐4‐phenyl‐1, 2, 3, 6‐tetrahydropyridine (MPTP)‐induced Parkinson's disease (PD) mice. (a) EcN‐GLP‐1 inhibited the microglia activation (Iba1), astrocyte activation (GFAP), and reduced the synaptic dysfunction (α‐syn) in the substantia nigra of PD mice. Representative results of immunohistochemical analysis (IHC) of the Iba1‐positive microglia, GFAP‐positive astrocytes, and the expression of α‐syn. Magnification ×400, Scale bar = 50 μm. (b) EcN‐GLP‐1 alleviated the reduction of tyrosine hydroxylase (TH)‐positive dopaminergic neuron numbers in the substantia nigra of PD mice. Representative results of immunofluorescence analysis (IF) of the TH‐positive cell numbers. Magnification ×200, scale bar = 100 μm. Quantification analysis of the content of Iba1 (c), GFAP (d), α‐Syn (e), and TH (f), which were related to the results of IHC and IF. C, The normal control group; M, the MPTP‐induced PD model group; ME, the EcN‐GLP‐1 treatment group; MG, the exenatide treatment group; MN, the EcN treatment group. Three mice of each group were randomly selected for analyzed, and data were presented as means ± SD. The significance of data was performed by Tukey's multiple Comparisons test, *p < 0.05, **p < 0.01, ***p < 0.001, ****p < 0.0001, ns indicates no significant difference (*p* > 0.05)

To observe whether EcN‐GLP‐1 could improve the neuropathological changes caused by MPTP stimulation, brain samples were collected for further analysis. Compared with the normal control mice, the number of GFAP‐positive astrocytes and Iba1‐positive microglia in the dense part of the substantia nigra increased remarkably in the PD mice (Figure 3a, c, and d). Moreover, the optical intensity of α‐Synuclein (α‐Syn), a kind of protein marker of PD, was enhanced obviously in the PD mice as well (Figure 3a and e), indicating that MPTP injection might lead to the higher expression of α‐Syn, and then cause brain injury. Noticeably, the treatments reversed those pathological changes in PD mice, particular under the administration of EcN‐GLP‐1 strain (Figure 3a). Meanwhile, the IF results showed that MPTP induction reduced the number of tyrosine hydroxylase (TH)‐positive neurons in the substantia nigra of PD mice, which would be alleviated by the treatments of EcN‐GLP‐1, exenatide, and EcN, with EcN‐GLP‐1 being the most efficient one (Figure 3b and f). Taken together, these results indicated that EcN combined with GLP‐1 could effectively alleviate the neuropathologic symptoms in MPTP‐induced mice.

### 3.3 EcN‐GLP‐1 inhibited brain inflammation in MPTP‐induced PD mice

**FIGURE 4:**
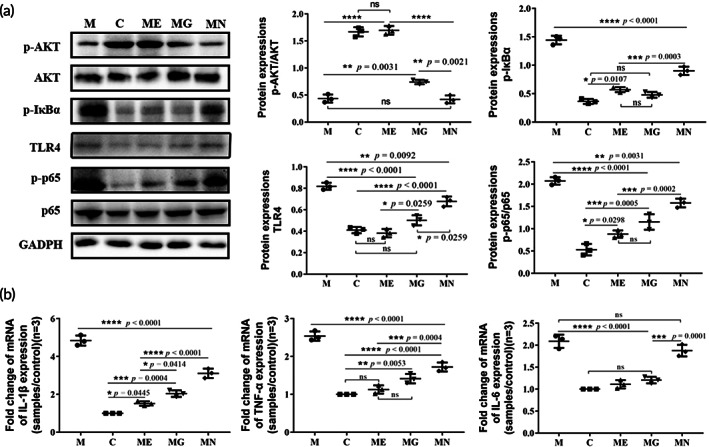
EcN‐GLP‐1 suppressed the neuroinflammation induced by 1‐methyl‐4‐phenyl‐1, 2, 3, 6‐tetrahydropyridine (MPTP) in Parkinson's disease (PD) mice. (a) Effects of EcN‐GLP‐1 on the expression of p‐AKT/AKT, p‐IκB‐α, TLR‐4, and p‐p65/p65 in the substantia nigra of MPTP‐induced PD mice. (b) EcN‐GLP‐1 reduced the pro‐inflammatory cytokines of TNF‐α, IL‐1β, and IL‐6 at gene level. C, The normal control group; M, the MPTP‐induced PD model group; ME, the EcN‐GLP‐1 treatment group; MG, the exenatide treatment group; MN, the EcN treatment group. Three mice were randomly selected for analyzed, and data were presented as means ± SD. Tukey's multiple Comparisons test, *p < 0.05, **p < 0.01, ***p < 0.001, ****p < 0.0001, ns indicates no significant difference (*p* > 0.05)

The AKT‐ and NF‐κB signaling pathways have been shown to play vital roles in the occurrence and progression of PD. To determine whether these pathways contributed to the treatment of PD by EcN‐GLP‐1, the expressions of some key proteins involved were evaluated. Compared to the normal mice, the intraperitoneal injection with MPTP led to a significant decrease the expression of p‐AKT/AKT, and an increase in the expression of p‐IκB‐α, TLR4, and p‐p65/p65 (Figure 4a). However, the administration of EcN‐GLP‐1 and exenatide could effectively enhance p‐AKT/AKT expressions while inhibiting that of p‐IκB‐α, TLR4, and p‐p65/p56. Notably, the treatment of EcN showed slight or no impact on the expression of those proteins, indicating that GLP‐1 and its analog were taking a leading position in regulating the expression of critical proteins induced by MPTP in PD mice (Figure 4a). Furthermore, MPTP modeling dramatically increased the transcriptional levels of some pro‐inflammatory cytokines, for example, IL‐1β, TNF‐α, and IL‐6, in the brain samples of PD mice compared with that of normal mice (Figure 4b). However, treatment using EcN‐GLP‐1 greatly mediated the most effective response of the host to the inflammatory conditions of PD than that of EcN, as EcN‐GLP‐1 could efficiently restore the mRNA expressions of those cytokines to the normal level (Figure 4b). In summary, the engineered probiotic strain of EcN‐GLP‐1 could inhibit the inflammatory response in the brain of MPTP‐induced PD mice via the combination of EcN and GLP‐1 expression.

### 3.4 EcN‐GLP‐1 attenuated inflammation and permeability in the colon of PD mice

**FIGURE 5:**
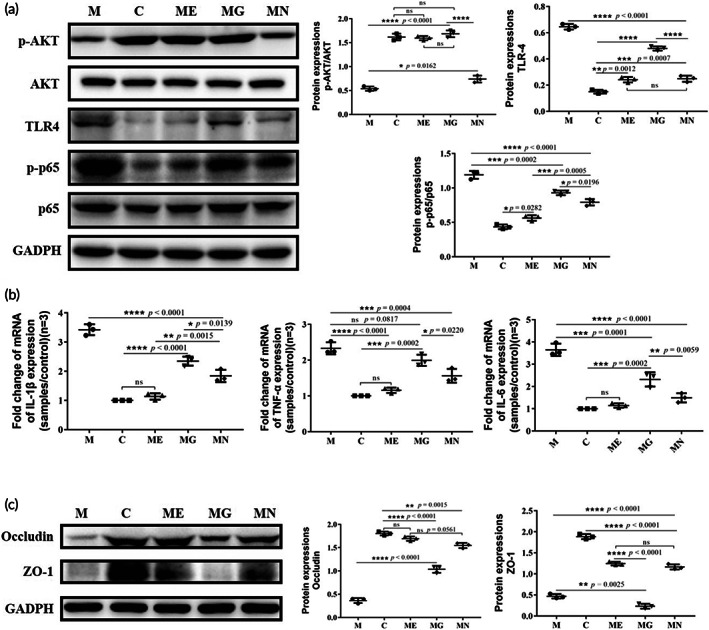
EcN‐GLP‐1 alleviated colonic inflammation and intestinal permeability in 1‐methyl‐4‐phenyl‐1, 2, 3, 6‐tetrahydropyridine (MPTP)‐induced Parkinson's disease (PD) mice. (a) Effects of EcN‐GLP‐1 on the expression of p‐AKT/AKT, TLR4, and p‐p65/p65 in the colon of PD mice. (b) EcN‐GLP‐1 reduced the pro‐inflammatory cytokines of TNF‐α, IL‐1β, and IL‐6 in the colon of PD mice. (c) EcN‐GLP‐1 increased the expression of ZO‐1 and occludin in the colon of PD mice. C, The normal control group; M, the MPTP‐induced PD model group; ME, the EcN‐GLP‐1 treatment group; MG, the exenatide treatment group; MN, the EcN treatment group. Three mice in each group were randomly selected for analyzed, and data were presented as means ± SD. Tukey's multiple Comparisons test, *p < 0.05, **p < 0.01, ***p < 0.001, ****p < 0.0001, ns indicates no significant difference (*p* > 0.05)

Emerging evidence suggests that inflammation in the intestine might be associated with pathogenesis in PD.^14^, ^34^ As shown in Figure 5a, the expression of p‐AKT/AKT was greatly reduced in the colon of MPTP‐induced PD mice; in contrast, TLR4 and p‐p65/p65 protein levels obviously increased compared to that of the normal mice. In comparison, the oral administration of EcN‐GLP‐1 could significantly reverse the changing trends of those proteins induced by MPTP with a more robust treatment effect than that of EcN and exenatide. Similarly, EcN‐GLP‐1 also measurably reduced the levels of IL‐1β, IL‐6, and TNF‐α, which were all stimulated expression in the colon of PD mice (Figure 5b). The chronic inflammatory condition in the colon of PD mice could break the integrity of the intestinal mucosal barrier and then increase the permeability of intestinal mucosa. In the colon of MPTP‐induced PD mice, the content of the tight junction proteins (TJs), mainly occludin and ZO‐1, had declined considerably (Figure 5c). Treatment with EcN‐GLP‐1 increased the levels of occludin and ZO‐1 compared to that of the MPTP‐induced mice, and similar results in EcN and exenatide groups were observed (Figure 5c). These results suggested that the constructed probiotic strain of EcN‐GLP‐1 could repair the damage to the intestinal barrier in MPTP‐induced PD mice by modulating the expression of TJs and inhibiting the inflammatory response in the NF‐κB signaling pathway.

### 3.5 EcN‐GLP‐1 improved gut microbiota dysbiosis in the MPTP‐induced PD mice

**FIGURE 6:**
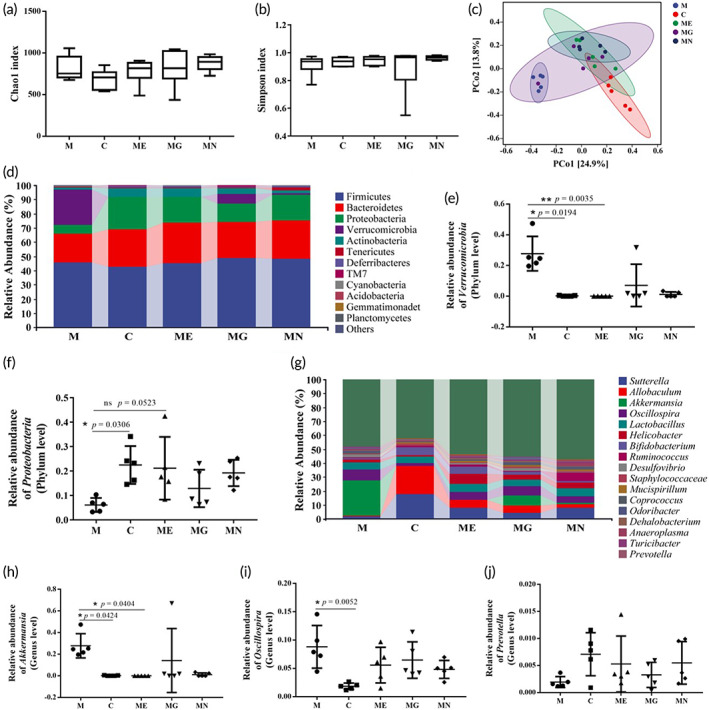
EcN‐GLP‐1 improved 1‐methyl‐4‐phenyl‐1, 2, 3, 6‐tetrahydropyridine (MPTP)‐induced gut microbiota dysbiosis in mice. EcN‐GLP‐1 had no impacts on the Chao1 (a) and Simpson (b) indices of gut microbiota. (c) PCoA analysis of gut microbiota. (d) Barplots of the relative abundance of gut bacteria at the phyla level. The relative abundance of *Verrucomicrobia* (e) and *Proteobactria* (f) at the phylum level. (g) Barplots of the relative abundance of gut bacteria at the genus level. The relative abundance of *Akkermansia* (h), *Oscillospira* (i), and *Prevotella* (j). C, The normal control group; M, the MPTP‐induced Parkinson's disease (PD) model group; ME, the EcN‐GLP‐1 treatment group; MG, the exenatide treatment group; MN, the EcN treatment group. Five fecal samples of each group were randomly selected for 16S rRNA sequencing and bioinformatic analysis. Data were presented as means ± SD. Multiple Comparisons test, *p < 0.05, **p < 0.01, ns and no asterisk indicate no significant difference (*p* > 0.05)

Accumulating evidence has revealed that both gut microbiota and microbial metabolites can affect the enteric nervous system (ENS) and the central nervous system (CNS) via the microbiota‐gut‐brain axis, suggesting intestinal dysbiosis might be closely associated with PD.^11^ Therefore, we used 16S rRNA sequencing combined with bioinformatic analysis to investigate the influence of EcN‐GLP‐1 on the microbial composition of MPTP‐induced mice. There were no significant differences in α‐diversity indices among these groups, for example, Chao1 index and Simpson index (Figure 6a,b). Moreover, the PCoA analysis showed that all samples in M group deviated significantly from those in other groups, whereas samples in ME, MN, and MG groups were closer to C group, with the exception of one sample of MG group that clustered in M group (Figure 6c).

The taxonomic classification analysis of OTUs derived from the primary sequencing data was performed to identify microbial community membership in the fecal samples of those five groups. At the phylum level, *Firmicutes*, *Bacteroidetes*, *Proteobacteria*, and *Verrucomicrobia* constituted the four most common dominant phyla in those five groups and accounted for 97.08%, 91.71%, 91.90%, 93.98%, and 94.55% of the total sequenced numbers of the five groups, respectively (Figure 6d). The relative abundance of *Verrucomicrobia* in M group was more abundant than that in the C group (*p* = 0.0194), while the relative level of *Proteobacteria* was less abundant compared with C group (*p* = 0.0306). Whereas, the relative abundance of *Verrucomicrobia* was reduced dramatically in the EcN‐GLP‐1 treated mice compared with M group (*p* = 0.0035), that of *Proteobacteria* was increased in the ME group (*p* = 0.0523) (Figure 6e,f). In addition, those treatments, including MPTP and EcN‐GLP‐1 had only a slight effect on the composition of *Firmicutes* and *Bacteroidetes*.

At the genus level, compared with C group, MPTP‐induced mice showed a decreased relative abundance of *Sutterella* (15.68% vs. 1.54%) and *Allobaculum* (20.12% vs. 0.69%), and an increased relative abundance of *Akkermansia* (0.20% vs. 25.14%) and *Oscillospira* (1.84% vs. 7.95%) (Figure 6g). Nevertheless, treatments with EcN‐GLP‐1, EcN, and exenatide, partly reversed this trend, including the changes of *Prevotella* (Figure 6h–j). These results suggested that the composition of gut microbiota could be altered after MPTP induction, but the use of EcN‐GLP‐1 restored the imbalance of the microbiome to a standard one.

## 4 DISCUSSION

Engineering microbial living therapeutics equipped with ingenious devices have been proved to perform specific actions in preventing and treating human diseases, which have been considered the next‐generation therapies.^26^ Recent advances in human commensal microbiota greatly broaden our vision of combining the productive and mutualistic features of gut microbiome into the living therapeutics. EcN, a kind of probiotic originally isolated from the gut, has been widely reported to have beneficial effects on gastrointestinal disorders,^35^ making it a preferred chassis for engineered living systems.^36^, ^37^, ^38^ Moreover, EcN has also been illustrated to reduce the development, progression, and severity of CNS inflammation by protecting the intestinal permeability in an experimental model.^39^

As we introduced above, although GLP‐1 showed beneficial actions in PD animal models and promising therapies in clinical trials,^6^ the short half‐life of GLP‐1 limits its clinical effect. Here, we chose EcN as the chassis to construct a recombinant integrant probiotic strain EcN‐GLP‐1 to deliver GLP‐1 molecule using CRISPR‐cas9 two plasmid system, an efficient gene‐editing tool of allelic exchange to generate genetically engineered *E. coli* strain.^27^, ^31^ We confirmed that this engineered probiotic strain EcN‐GLP‐1 could efficiently express and secrete GLP‐1 into the surrounding extracellular space without affecting its growth and probiotic properties (Figure 1). We also demonstrated that EcN‐GLP‐1 showed significant neuroprotective impacts on MPTP‐induced PD mice via producing GLP‐1 therapeutic molecule and modulating the disturbed intestinal dysfunctions compared to that of EcN alone.

Mice injected intraperitoneally with MPTP showed severe behavioral dysfunctions, including increased latency time, reduced total movement distance, and even death because of the high toxin of MPTP.^23^, ^40^ Our results also found that some of the mice died during the early stage of modeling, and the remaining mice showed motor deficits that were proved by pole test and open field test (Figure 2). However, the impaired motor function had been improved by administrating EcN‐GLP‐1, EcN, and exenatide, with EcN‐GLP‐1 showing the best treatment effect (Figure 2).

PD has been characterized by the accumulation of α‐Syn and the activation of astrocytes and microglia in the brain pathologically.^41^ To investigate the underlying mechanisms of EcN‐GLP‐1 in treating PD, IHC, and IF assays were performed to detect the expression of essential proteins involved in the pathology of PD, such as TH, Iba1, GFAP, and α‐Syn (Figure 3). The MPTP‐induced mice showed increased Iba1‐positive microglia and GFAP‐positive astrocytes in the brain samples, proving an activation of astrocytes and microglia surrounding the degenerated neurons in the SN of PD.^42^ Moreover, α‐Syn, a characteristic protein of PD aggregated and misfolded in cells of PD patients,^43^ was enhanced in the MPTP‐induced mice as well. In contrast, MPTP induction decreased the expression of TH, a rate‐limiting enzyme that synthesizes dopamine, which is reduced or even depleted in the SN along with the progressively damaged dopaminergic neurons.^44^ Compared with the M and EcN groups, EcN‐GLP‐1, and exenatide treatments significantly reversed those changes, suggesting that supplementation of GLP‐1 through engineered EcN‐GLP‐1 strain or its analog might protect the degeneration of dopaminergic neurons by modulating the content of major protein involved in PD‐associated multiple cellular pathways.

MPTP can be converted into the toxic form of MPP^+^; the latter would activate NF‐κB in microglia and astrocytes, and then facilitate the release of pro‐inflammatory cytokines, promoting the PD progression with α‐Syn dysfunction.^41^, ^45^ In response to the neuroinflammatory environment, GLP‐1 will cross the blood–brain barrier (BBB), and bind to its receptor (GLP‐1R), which is selectively localized in neurons throughout the brain with high densities in the SN.^46^, ^47^ Subsequently, the binding of GLP‐1 with GLP‐1R further triggers the PI3K/protein kinase B (AKT) pathway, and in turn induces the expression of IκBα and inhibits the downstream of NF‐κB signaling pathway to suppress the expression of pro‐inflammatory cytokines.^48^, ^49^ Our results demonstrated that the expression of p‐AKT/AKT was suppressed dramatically in the brain tissues of MPTP‐induced mice, while the expression of p‐IκB‐α, TLR4, p‐p65/65, and common pro‐inflammatory cytokines (IL‐6, IL‐1β, TNF‐α) were upregulated obviously (Figure 4). These results suggested that the neuroinflammation is associated with the neurodegeneration of PD.^8^, ^48^ Our results also illustrated that these inflammation responses could be alleviated in the EcN‐GLP‐1 and exenatide groups, and noticeably, using EcN alone just showed no or slightly actions in regulating these inflammatory pathways in PD (Figure 4).

Emerging data suggest that intestinal inflammation contributes to the pathogenesis of PD.^14^, ^34^ Our data also suggested that the MPTP‐induced PD mice had an obvious inflammatory condition in the colon, mainly in the increased expression of TLR‐4, p‐p65/p65, and pro‐inflammatory cytokines including IL‐1β, IL‐6 and TNF‐α, and suppressed p‐AKT/AKT (Figure 5). When orally administrated with EcN and EcN‐GLP‐1, the level of colon inflammation dropped significantly compared to that of exenatide, which means that the better therapeutic effects might benefit more from the probiotic properties of EcN.^35^, ^39^ Tight junction proteins such as ZO‐1 and occludin play a vital role in maintaining the intestinal barrier to prevent the invasion of gut pathogens into immune cells under the intestinal mucosa.^35^ Furthermore, our results indicated that an impaired intestinal epidermal barrier function that would further increase mucosal permeability,^11^, ^50^ was emerged in the colon of MPTP‐induced PD mice,^51^ which could be improved obviously by EcN‐GLP‐1 and EcN (Figure 5). Interestingly, exenatide treatment alone showed a moderate impact on reversing the expression of occludin and no effect on that of ZO‐1. Collectively, the engineered probiotic strain EcN‐GLP‐1 had actions in alleviating the intestinal inflammation in PD mice by inhibiting the NF‐κB pathway and repairing the intestinal permeability.

Several observations have confirmed that the gut microbiota might be a risk factor for the development of PD, accompanied by an alteration of gut microbial structure and composition in patients and animal models of PD.^49^, ^52^ In this work, the abundances of several intestinal bacteria, including *Verrucomicrobia*, *Akkermansia*, and *Oscillospira*, were increased, and that of *Prevotella* was decreased during the progress of PD in mice (Figure 6), which are consistent with the results of other studies.^53^, ^54^, ^55^, ^56^ Furthermore, the alteration of those bacteria could be restored by treating with EcN‐GLP‐1, indicating that the engineered EcN‐GLP‐1 strain could promote the conversion of the disturbed gut microbiota in PD mice to the standard one (Figure 6). Among them, *Akkermansia* may directly expose the intestinal plexus to oxidative stress, leading to degradation of the intestinal mucus layer and abnormal accumulation of α‐Syn in the intestine,^12^, ^49^, ^53^ while *Prevotella* is efficient in producing short‐chain fatty acids by metabolizing nutrients, the latter has been proven to enhance gastrointestinal motility, exert anti‐inflammatory effects and trigger GLP‐1 secretion in mixed colonic cell cultures in vitro.^53^, ^57^

## 5 CONCLUSION

**FIGURE 7:**
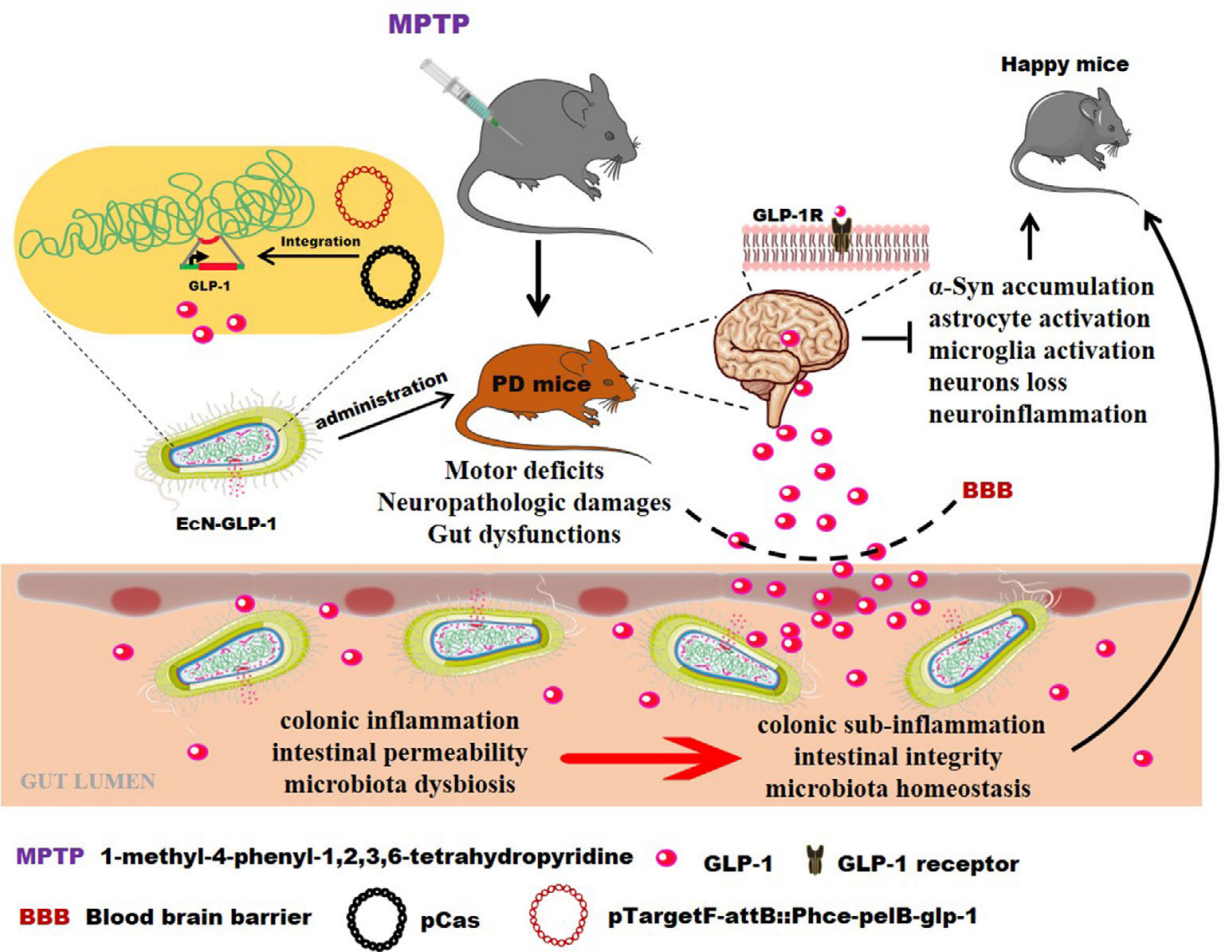
Schematic diagram of the underlying mechanisms of EcN‐GLP‐1 in improving Parkinson's disease (PD). EcN‐GLP‐1 exerted neuroprotective effects against neurodegeneration and intestinal dysbiosis in 1‐methyl‐4‐phenyl‐1, 2, 3, 6‐tetrahydropyridine (MPTP)‐induced PD mice via delivering GLP‐1 and modulating gut microbiota. GLP‐1, glucagon‐like peptide‐1; EcN‐GLP‐1, the engineered *Escherichia coli* Nissle 1917 that GLP‐1 cluster genes has been recombinantly integrated into its chromosome

Here, we constructed a recombinant integrant *E. coli* Nissle strain to deliver GLP‐1 and assessed its neuroprotective effects on the MPTP‐induced PD mice. Our evidence elucidated that the engineered probiotic strain EcN‐GLP‐1 showed effective neuroprotection in PD mice by attenuating the associated inflammation and adjusting the intestinal dysbiosis. EcN‐GLP‐1 could persistently produce and deliver active GLP‐1 drug molecule that can cross the BBB and bind to its receptors localized in neurons throughout the brain, which would activate the pathway of GLP‐1/GLP‐1R to exert the therapeutic effects on PD (Figure 7). Furthermore, EcN‐GLP‐1 could also combine the advantages of the chassis EcN to modulate the gut microbiota dysbiosis to strengthen its beneficial efficacy in treating PD (Figure 7). However, this study had some limitations due to lacking the dose dependence and unidentified the precise mechanisms of EcN‐GLP‐1 in improving PD. Therefore, we propose that the properties and roles of this strain should be further evaluated in gut‐brain conditions, including PD in the future.

## AUTHOR CONTRIBUTIONS

**Heng Wu:** Data curation (equal); investigation (equal); methodology (equal); writing – original draft (equal). **Jing Wei:** Investigation (equal); methodology (equal); supervision (equal); validation (equal). **Xiumiao Zhao:** Investigation (equal); writing – original draft (equal). **Ying Liu:** Data curation (equal); investigation (equal); validation (equal). **Zhihang Chen:** Data curation (equal); investigation (equal); writing – original draft (equal). **Kehong Wei:** Investigation (equal); validation (equal). **Jiachen Lu:** Investigation (equal); validation (equal). **Wenjie Chen:** Investigation (equal); validation (equal). **Meixiu Jiang:** Methodology (equal); validation (equal). **Shengjie Li**: Investigation (equal); supervision (equal); validation (equal); writing – original draft (lead); writing – review and editing (lead). **Tingtao Chen:** Conceptualization (lead); funding acquisition (lead); project administration (lead); writing – review and editing (equal).

## CONFLICT OF INTERESTS

The authors declare no conflict of interest.

6

### PEER REVIEW

The peer review history for this article is available at https://publons.com/publon/10.1002/btm2.10351.

## Supporting information


**Data S1** Supplementary Information
**Table S1.** Stains and plasmids used in this experiment
**Table S2.** Primers used in this study
**Figure S1.** The design and procedure of the CRISPR‐Cas9 two‐plasmid system, related to Section 2.2. (A) Schematic view of pCas and recombinant pTarget‐*attB*::Phce‐pelB‐glp‐1 plasmids, (B) Diagram of integration of GLP‐1 cluster genes into the chromosome of EcN.
**Figure S2.** PCR analysis to confirm the integration of GLP‐1 cluster genes into the chromosome of EcN strain. (A) Detection of the upstream of *attB* (lane 1, P3/P4 primers), the codon‐optimized GLP‐1 gene clustered with *HCE* promotor and *pelB* signal peptide sequences (lane 2, P5/P6 primers), and the downstream of *attB* (lane 3, P7/P8 primers), (B) The result of overlapped‐PCR (lane1, P3/P8 primers), (C) Colony PCR to confirm the integrated strain (lane 1) and EcN strain (lane 2) using P9/P10 primers, (D) PCR analysis to further confirm the recombinant integrant probiotic strain using P13/P14 primers.

## Data Availability

Data supporting this study are available from the corresponding authors on reasonable request.
