# Supplementary material for: Neuroprotective effects of an engineered Escherichia coli Nissle 1917 on Parkinson's disease in mice by delivering GLP‐1 and modulating gut microbiota
Source: Bioeng Transl Med. 2022 Jun 18;8(5):e10351. doi: 10.1002/btm2.10351 (PMC10487327; doi:10.1002/btm2.10351)
Supplement: Supplementary file 1 — Data S1 Supplementary Information Table S1. Stains and plasmids used in this experiment Table S2. Primers used in this study Figure S1. The design and procedure of the CRISPR‐Cas9 two‐plasmid system, related to Section 2.2. (A) Schematic view of pCas and recombinant pTarget‐attB::Phce‐pelB‐glp‐1 plasmids, (B) Diagram of integration of GLP‐1 cluster genes into the chromosome of EcN. Figure S2. PCR analysis to confirm the integration of GLP‐1 cluster genes into the chromosome of EcN strain. (A) Detection of the upstream of attB (lane 1, P3/P4 primers), the codon‐optimized GLP‐1 gene clustered with HCE promotor and pelB signal peptide sequences (lane 2, P5/P6 primers), and the downstream of attB (lane 3, P7/P8 primers), (B) The result of overlapped‐PCR (lane1, P3/P8 primers), (C) Colony PCR to confirm the integrated strain (lane 1) and EcN strain (lane 2) using P9/P10 primers, (D) PCR analysis to further confirm the recombinant integrant probiotic strain using P13/P14 primers. [file BTM2-8-e10351-s001.docx]

**Supporting information**

**Neuroprotective effects of an engineered *E. coli* Nissle on Parkinson's disease in mice by delivering GLP-1 and modulating gut microbiota**

Heng Wu^1, #^, Jing Wei^1, #^, Xiumiao Zhao^2^, Ying Liu^3^, Zhihang Chen^2^, Kehong Wei^2^, Jiachen Lu^2^, Wenjie Chen^2^, Meixiu Jiang^1^, Shengjie Li ^1,^ * and Tingtao Chen^1,^ *

^1^ National Engineering Research Center for Bioengineering Drugs and the Technologies, Institute of Translational Medicine, Nanchang University, Nanchang, China

^2^ Queen Mary School, Nanchang University, Nanchang, China

^3^ Institute of Life Science, Nanchang University, Nanchang, China

^#^ Contribute equally to this work.

^*^ Correspondence: Tingtao Chen and Shengjie Li, Institute of Translational Medicine, Nanchang University, 1299 University Avenue, Nanchang 330031, China.

Email: [chentingtao1984@163.com](mailto:chentingtao1984@163.com) and [lishengjie1104@ncu.edu.cn](mailto:315794984@qq.com)

| **Supplementary Table 1.** Stains and plasmids used in this experiment | | |
| --- | --- | --- |
| strain and plasmid | Relevant feature | reference or source |
| Strains |  |  |
| EcN | *E. coli* Nissle 1917 strain | Laboratory stock |
| EcN-GLP-1 | EcN integrated with GLP-1 gene under HCE promotor and pelB signal peptide in the attB locus in the chromosome of EcN | This study |
| *Trans*5α | F^-^φ80d lacZΔM15 Δ(lacZYA-argF) U169 end A1 recA1 hsdR17 (r_k_^-^, m_k_^+^) supE44λ- thi-1 gyrA96 relA1 phoA | TransGen Biotech Beijing, China |
| *S. aureus* | *Staphylococcus aureus* Cowan1 | Laboratory stock  Wang et al., 2020 |
| *S. typhimurium* | *Salmonella typhimurium* ATCC 13311 |  |
| *S. enteritidis* | *Salmonella enteritidis* ATCC 13076 |  |
|  |  |  |
| Plasmids |  |  |
| pCas | repA101(Ts) kan Pcas-cas9 ParaB-Red lacIq Ptrc-sgRNA-pMB1 | Jiang et al., 2015 |
| pTargetF | pMB1 aadA sgRNA |  |
| pBSC-pelb-hglp | Codon-optimized GLP-1 gene under Phce promotor and pelB signal peptide sequences were synthesized and coloned in this plasmid | Laboratory stock |
| pTargetF-*attB* | pTargetF infused with the N20 sequence of attB target loci | This study |
| pTargetF-attB::P_hce_-pelB-glp-1 | pTargetF-attB infused with GLP-1 cluster genes | This study |

| **Supplementary Table 2.** Primers used in this study | |
| --- | --- |
| Primers for PCR amplification and sequencing | sequence (5'-3') |
| P1 | AGTCCTAGGTATAATACTAGTTCAAGTTAGTATAAAAAAGCGTTTTAGAGCTAGAAATAG |
| P2 | ACTAGTATTATACCTAGGACTGAGCTAGCTGTCAAG |
| P3 | ttctctagagtcgacctgcagGAAAGCCCAATCTTCACATCAATC |
| P4 | CCTGTGTGAAATTGTTATCCGCTAAAAAAGCAGGCTTCAAC |
| P5 | TGAAGCCTGCTTTTTTAGCGGATAACAATTTCACACAGGA |
| P6 | CGCTCAAGTTAGTATCCCAGTCACGACGTTGTAAAACGAC |
| P7 | ACAACGTCGTGACTGGGATACTAACTTGAGCGAAACGGGA |
| P8 | cagggtaatagatctaagcttTTACGATGGCGATAATATTTCACC |
| P9 | AAGCTTAGATCTATTACCCTGT |
| P10 | CTGCAGGTCGACTCTAGAGAA |
| P11 | TGCGCCGCGACCAGAAACGAT |
| P12 | CGTAGTACGCATCGGTACGCC |
| P13 | CAGGAAACAGCTATGAC |
| P14 | ACTGGCCGTCGTTTTAC |
| 515F | GTGCCAGCMGCCGCGGTAA |
| 806R | GGACTACHVGGGTWTCTAAT |
| Primers for RT-qPCR | |
| IL-6-F | CTTCTTGGGACTGATGCTGGTGAC |
| IL-6-R | AGGTCTGTTGGGAGTGGTATCCT |
| TNF-α-F | GTGGAACTGGCAGAAGAGGCA |
| TNF-α-R | AGAGGGAGGCCATTTGGGAAC |
| IL-1β-F | GTGTCTTTCCCGTGGACCTTC |
| IL-1β-R | TCATCTCGGAGCCTGTAGTGC |
| GAPDH-F | CTCGTGGAGTCTACTGGTGT |
| GAPDH-R | GTCATCATACTTGGCAGGTT |
| N20 sequences and PAM of attB target loci | |
| attB | TCAAGTTAGTATAAAAAAGCAGG |

Supplementary Figure 1


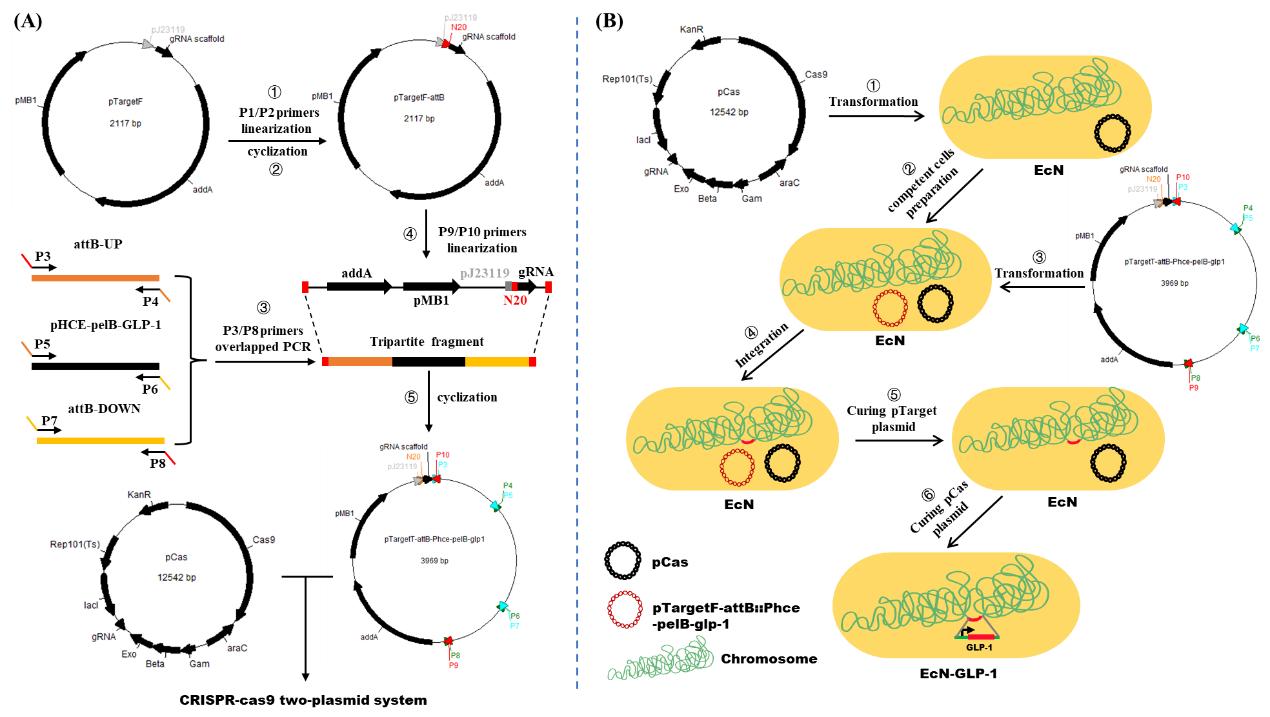


**FIGURE S1** The design and procedure of the CRISPR-Cas9 two-plasmid system, related to section 2.2. (A) Schematic view of pCas and recombinant pTarget-*attB*::Phce-pelB-glp-1 plasmids, (B) Diagram of integration of GLP-1 cluster genes into the chromosome of EcN.

Supplementary Figure 2


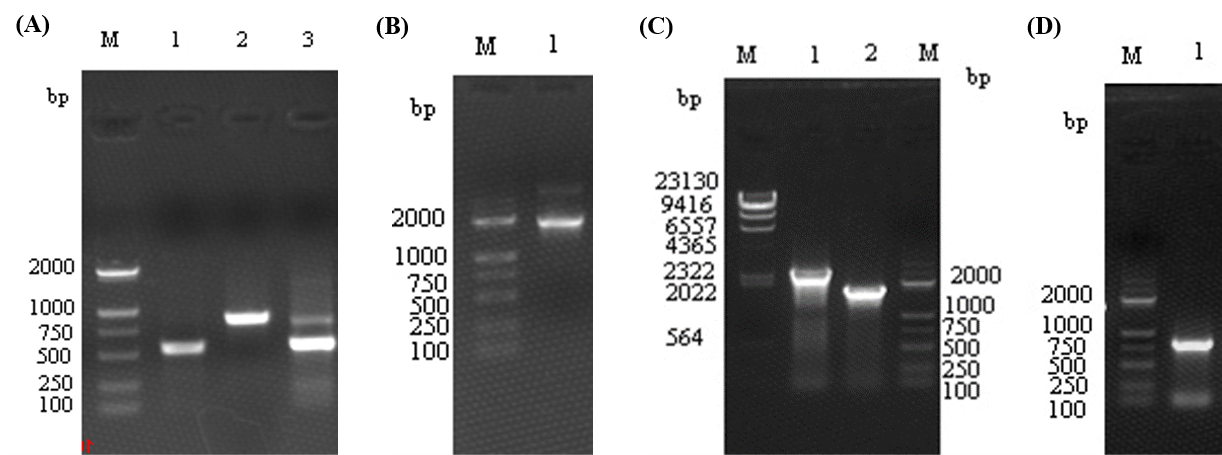


**FIGURE S2** PCR analysis to confirm the integration of GLP-1 cluster genes into the chromosome of EcN strain. (A) Detection of the upstream of *attB* (lane 1, P3/P4 primers), the codon-optimized GLP-1 gene clustered with *HCE* promotor and *pelB* signal peptide sequences (lane 2, P5/P6 primers), and the downstream of *attB* (lane 3, P7/P8 primers), (B) The result of overlapped-PCR (lane1, P3/P8 primers), (C) Colony PCR to confirm the integrated strain (lane 1) and EcN strain (lane 2) using P9/P10 primers, (D) PCR analysis to further confirm the recombinant integrant probiotic strain using P13/P14 primers.
